# An insight into small extracellular vesicles: Their roles in colorectal cancer progression and potential clinical applications

**DOI:** 10.1002/ctm2.249

**Published:** 2020-12-12

**Authors:** Xuefeng He, Xinyang Zhong, Zijuan Hu, Senlin Zhao, Ping Wei, Dawei Li

**Affiliations:** ^1^ Department of Colorectal Surgery Fudan University Shanghai Cancer Center Shanghai China; ^2^ Department of Pathology Fudan University Shanghai Cancer Center Shanghai China; ^3^ Cancer Institute Fudan University Shanghai Cancer Center Shanghai China; ^4^ Institute of Pathology Fudan University Shanghai China; ^5^ Department of Oncology Shanghai Medical College Fudan University Shanghai China

**Keywords:** Cancer Metastasis, Cancer Therapy and Diagnosis, Colorectal Cancer, Extracellular Vesicles, Small Extracellular Vesicles

## Abstract

Colorectal cancer (CRC) is one of the most common cancers and a leading cause of mortality worldwide. Small extracellular vesicles (sEVs) are nano‐sized extracellular vesicles containing a variety of bioactive molecules, such as nucleic acids, proteins, lipids, and metabolites. Recent evidence from CRC has revealed that sEVs contribute to tumorigenesis, progression, and drug resistance, and serve as a tool for “liquid biopsy” and a drug delivery system for therapy. In this review, we summarize information about the roles of sEVs in the proliferation, invasion, migration, epithelial‐mesenchymal transition, formation of the premetastatic niche, and drug resistance to elucidate the mechanisms governing sEVs in CRC and to identify novel targets for therapy and prognostic and diagnostic biomarkers.

## Graphical abstract

Colorectal cancer (CRC) is the third most commonly diagnosed cancer worldwide and patients with metastasis usually have a poor prognosis. Small extracellular vesicles (sEVs) have been identified to play a significant role in intercellular communication. sEVs released from different cells exert huge effects on CRC cell proliferation and metastasis. They could also serve as biomarkers for CRC diagnosis and prognosis and be a potential drug delivery system to treat CRC patients.

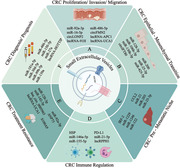

**Abbreviations**
BMDCs: bone marrow‐derived dendritic cells
BMSCs: bone marrow‐derived mesenchymal stem/stromal cells
CAF: cancer‐associated fibroblast
CCL: CC chemokine ligand
ceRNAs: competing endogenous RNAs
CRC: colorectal cancer
CSC: cancer stem cell
CTCs: circulating tumor cells
DCs: dendritic cells
EGFR: epidermal growth factor receptor
EMT: epithelial‐mesenchymal transition
ESCRT: endosomal sorting complex required for transport
ESEs: early‐sorting endosomes
EVs: extracellular vesicles
HSP: heat shock protein
ILVs: intraluminal vesicles
ITG: integrin
ITGA2: integrin α2
KLF: Krüppel‐like factor
LARC: locally advanced rectal cancer
LN: lymph node
miRNAs: microRNAs
MVBs: multivesicular bodies
ncRNAs: noncoding RNAs
NETs: neutrophil extracellular traps
PD‐L1: programmed cell death ligand‐1
PKM: pyruvate kinase
PMN: premetastatic niche
PTEN: phosphatase and tensin homolog deleted on chromosome ten
Rab: Ras­related proteins in brain
sEVs: small extracellular vesicles
TAMs: tumor‐associated macrophages
TGF‐β: transforming growth factor‐β
TLR: Toll‐like receptor
TME: tumor microenvironment
TSG101: tumor susceptibility 101

## 1 BACKGROUND

Colorectal cancer (CRC) is the third most commonly diagnosed cancer worldwide, while the death rate ranks second, with approximately 900 000 deaths recorded annually.^1^ CRC is generally asymptomatic at the early stage, which highlights the importance of early detection and diagnosis.^2^ Although screening approaches have been implemented throughout the world, unfortunately, the early diagnosis rate of CRC only reaches 30‐40%, which is far from our goal.^3^ Although therapies, including the application of laparoscopy, chemotherapy, radiotherapy, and target agents, have rapidly developed in recent years, the prognosis of patients with CRC generally remains poor,^4^ with a 5‐year survival rate of only 10% for patients with advanced stage tumors or with metastasis.^5^ Hence, the development of suitable and measurable biomarkers for an early diagnosis and predicting the prognosis has attracted increasing attention.

Extracellular vesicles (EVs) used to be identified as two main subtypes based on the mechanisms of biogenesis: endosome‐origin small extracellular vesicles (sEVs) and plasma membrane‐derived ectosomes (microvesicles/microparticles),^6^ with a diameter fluctuating from 50 to 1000 nm and from 40 to 160 nm, respectively.^7^ Over the past few decades, EVs have spawned great interest of their role in the progression of various cancers and their clinical potential. In view of the large number of researches of CRC focus in recent years, we will mainly summarize a specific subset of EVs referring to “exosomes” in publications. However, with an increasing understanding of EVs, it is unambiguous that the ultimate origin of vesicles cannot be discriminated yet. And it is unavoidable that most methods applied to isolate exosomes so far will coisolate heterogeneous EVs from diverse origin. As a result, based on the nomenclature illustrated in the Minimal Information for Studies on Extracellular Vesicles 2018 (MISEV2018) published by the International Society for Extracellular Vesicles (ISEV),^8^ we will apply the terminology “small extracellular vesicles” (sEVs) (diameter <200 nm or <100 nm) in place of “exosomes” (Tables 1 and 2).

**TABLE 1:** Main types of extracellular vesicles

Vesicles	Size (nm)	Origin	Reference
Exosomes	40‐160	endosomes	^7^
Microvesicles	100‐1000	Plasma membrane	^8^
Apoptotic bodies	500‐2000	Plasma membrane, endoplasmic reticulum	^158^

**TABLE 2:** MISEV2018‐recommended nomenclature^8^

Characteristics	Recommended nomenclature	
Physical characteristics	Size	Small: diameter <200 nm or <100 nm Large and/or medium: >200 nm
	Density	Low; middle; high
Biochemical composition	eg, CD63+/CD81+ EVs, PD‐L1+ EVs, etc	
Conditions or cell of origin	eg, Apoptotic EVs, hypoxic EVs, etc	

Although sEVs were initially underestimated as “cellular debris” and a system to dispose of cellular components when they were first discovered in 1983 by two independent groups, now they are considered to play a significant role in intercellular communication.^7^, ^9^, ^10^ Based on emerging evidence, biomolecules loaded in sEVs are shuttled to recipient cancer cells or stromal cells, thus modulating their activity by regulating signaling pathways. sEVs are involved in tumor proliferation, metastasis, angiogenesis, immune regulation, and even drug resistance.^11^ Additionally, cancer cell‐derived sEVs carry a number of cancer‐specific biomolecules, such as proteins, microRNAs (miRNAs), and lncRNAs, which might serve as biomarkers for the early detection of CRC.^12^, ^13^ In this review, we summarize the biogenesis of sEVs, their roles in CRC progression (Table 3), and their promising clinical applications (Table 4).

**TABLE 3:** The role of the substances in CRC sEVs

Cargo	Parent cell	Recipient cell	Pathway and target	Biofunction	Reference
**MiRNAs**					
miR‐21	CAFs	CRC cells	/	Promote proliferation, invasion, and metastasis	^159^
miR‐92a‐3p	CAFs	CRC cells	FBXW7 and MOAP1/Wnt/β‐catenin	Promote invasion and metastasis	^29^
miR‐486‐5p	CRC cells	CRC cells	PLAGL2/IGF2/β‐catenin	Promote proliferation, invasion, and metastasis	^30^
miR‐193a	CRC cells	CRC cells	/	Promote proliferation, invasion, and metastasis	^160^
miR‐16‐5p	BMSCs	CRC cells	ITGA2	Inhibit proliferation, invasion, metastasis, and promote apoptosis	^33^
miR‐128‐3p	CRC cells	CRC cells	Bmi1	EMT	^113^
miR‐106b‐3p	CRC cells	CRC cells	DCL‐1	Promote invasion, metastasis, and EMT	^44^
miR‐25‐3p miR‐130b‐3p miR‐425‐5p	CRC cells	TAMs	PTEN/PI3K/Akt/STAT6	EMT	^45^
miR‐25‐3p	CRC cells	Endothelial cells	KLF/ZO‐1 and occluding and Claudin5	PMN and metastasis	^45^
miR‐200s	CRC cells	Endotheliocytes	/	EMT	^42^, ^43^
miR‐21	CRC cells	TAMs	TLR7/IL‐6	PMN	^47^
miR‐1229	CRC cells	Endothelial cells	HIPK2/MEF2C/VEGF	Angiogenesis	^59^
miR‐146a‐5p	CSCs	CD8^+^ T cells	/	Promote immunosuppressive microenvironment	^80^
miR‐1246	GOF mutp53 cancer cells	Macrophages	TGF‐β	Inhibit macrophage polarization	^67^
miR‐203	CRC cells	Monocytes	/	Promote metastasis	^161^
miR‐21‐5p miR‐155‐5p	M2 TAMs	CRC cells	BRG1	Promote migration, invasion, and metastasis	^87^
**LncRNAs**					
LncRNA 91H	CRC cells	CRC cells	HNRNPK	Promote invasion and metastasis	^37^
LncRNA UCA1	CRC cells	CRC cells	miR‐143/MYO6	Promote proliferation, apoptosis, and metastasis	^38^
LncRNA APC1	CRC cells	Endothelial cells	p38‐MAPK	Angiogenesis	^32^
LncRPPH1	CRC cells	Macrophages	/	Promote proliferation and metastasis	^86^
**CircRNAs**					
CircIFT80	CRC cells	CRC cells	miR‐1236‐3p/HOXB7	Promote proliferation, metastasis, and EMT	^46^
CircFMN2	CRC cells	CRC cells	miR‐1182/hTERT	Promote proliferation	^39^
CircLONP2	CRC cells	CRC cells	miR‐17	Promote invasion and metastasis	^40^
**Others**					
Wnt4	CRC cells	CRC cells	Wnt/β‐catenin	Promote proliferation and metastasis	^28^
CCL2	CRC cells	TAMs	/	PMN	^69^
Integrins (ITGs)	CRC cells	CAFs	/	PMN	^74^
PD‐L1	CRC cells	T cells	/	Promote proliferation and drug resistance	^78^
CXCL1, CXCL2	CRCSC	Neutrophils	IL‐1β	Immune regulation	^94^
HSP	CRC cells	NK cells	Granzyme B	Initiate apoptosis	^99^

**TABLE 4:** The clinical application of CRC sEVs components

Cargo	Parent cell	Source of sEVs	Biomarker potential	Reference
**MiRNAs**				
miR‐181a‐5p	Hypoxic tumor cells	Plasma	Prognosis	^141^
miR‐486‐5p	Hypoxic tumor cells	Plasma	Prognosis	^141^
miR‐30d‐5p	Hypoxic tumor cells	Plasma	Prognosis	^141^
miR‐150‐5p	CRC cells	Serum	Diagnosis and prognosis	^143^
miR‐99b‐5p	CRC cells	Serum	Diagnosis and prognosis	^143^
miR‐27a	CRC cells	Plasma	Diagnosis and prognosis	^144^
miR‐130a	CRC cells	Plasma	Diagnosis and prognosis	^144^
miR‐92b	CRC cells	Plasma	Diagnosis	^162^
miR‐122	CRC cells	Serum	Diagnosis and prognosis	^163^
miR‐424‐5p	CRC cells	Serum	Diagnosis	^164^
**LncRNAs**				
LNCV6_116109/LNCV6_98390/LNCV6_38772/LNCV_108266/LNCV6_84003/LNCV6_98602	CRC cells	Plasma	Diagnosis (stages I‐II)	^3^
HOTTIP	CRC cells	Serum	Prognosis	^142^
LINC02418	CRC cells	Serum	Diagnosis	^165^
**CircRNAs**				
hsa‐circ‐0004771	CRC cells	Serum	Diagnosis	^166^
circ‐PNN	CRC cells	Serum	Diagnosis	^167^

## 2 sEVs: BIOGENESIS AND CHARACTERIZATION

**FIGURE 1:**
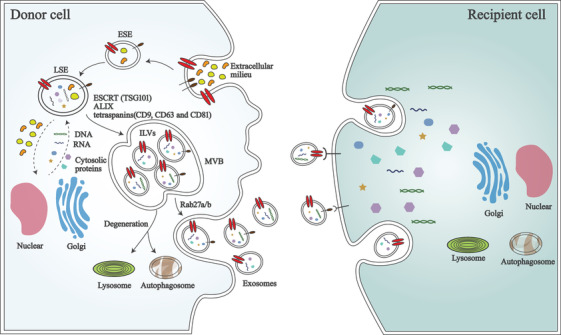
The main procedure of exosomes biogenesis and release. Cellular plasma membrane invaginates to form early‐sorting endosomes (ESEs), then ESEs mature into late‐sorting endosomes (LSEs), and inward budding of endosomal membrane results in multivesicular bodies (MVBs). Tumor susceptibility 101 (TSG101), ALG‐2 interacting protein X (ALIX), and tetraspanins (CD9, CD63, and CD81) are indispensable parts in endosomal sorting complex required for transport (ESCRT)‐dependent way in the process of MVBs biogenesis. MVBs fuse with cellular plasma membrane to release intraluminal vesicles (ILVs) as exosomes. Rab27a is associated with membrane fusion and endosomal size, whereas Rab27b is connected with exosomes redistribution. After secretion, exosomes uptaken by recipient cells could be mediated by endocytosis, fusion with the plasma membrane, or ligand/receptor interaction

In this part, we only describe the biogenesis of exosomes because the process of exosomes is most well illustrated. It primarily occurs in several steps, as described below. (a) The formation of early‐sorting endosomes (ESEs): The invagination of the cellular plasma membrane leads to the formation of ESEs, which contain proteins from the cell surface and extracellular milieu. (b) The formation of multivesicular bodies (MVBs): After ESEs mature into late‐sorting endosomes, the inward budding of the endosomal membrane results in the formation of MVBs. (c) The generation of exosomes: MVBs, which are characterized by the presence of intraluminal vesicles (ILVs), either fuse with the cellular plasma membrane to release the ILVs as exosomes containing specific biomolecules into the extracellular space or fuse with lysosomes or autophagosomes to be degraded^7^ (Figure 1).

Many researchers have attempted to explore the mechanisms of exosomes’ formation. The endosomal sorting complex required for transport (ESCRT) consists of four main complexes (ESCRT‐0, I, II, and III) and is known to be the most important machinery responsible for delivering specific molecules into ILVs of the MVBs and for protein recycling. ESCRT‐0, which is activated by phosphatidylinositol 3‐phosphate, recruits ESCRT‐I to form the ESCRT‐I complex by interacting with tyrosine kinase substrate prosaposin domains and ESCRT‐I subunit tumor susceptibility 101 (TSG101). The complex is necessary for sorting cargo and transferring ubiquitinated transmembrane proteins into the MVBs. ESCRT‐II is involved in the initiation of inward budding processes. ESCRT‐III is regulated by complexes I and II, and plays key roles in cargo sorting and concentration, vesicle scission, and protein recycling. Additionally, ALG‐2 interacting protein X, an accessory protein of ESCRT, functions in the ESCRT pathway to regulate the process of exosomes biogenesis.^14^

In addition to the ESCRT‐dependent pathway, an ESCRT‐independent mechanism also exists, and some proteins, such as the Ras­related proteins in brain (Rab) family (Rab27a and Rab27b), tetraspanins (CD9, CD63, and CD81), and sphingomyelinase, have been shown to engage in membrane fusion, endosomal vesicle trafficking, and vesicle release. Among these proteins, Rab27a is associated with membrane fusion and the endosomal size, whereas Rab27b knockdown redistributes multivesicular endosomes to the perinuclear region. However, researchers should focus on elucidating the precise mechanism of the ESCRT‐independent pathway, due to the insufficient understanding of the mechanism.^15^

The composition of double‐layered and cup‐shaped sEVs is heterogeneous and reflects their cellular origin and physiological and pathological states. Because sEVs are synthesized by most cell types, such as T lymphocytes, B cells, and dendritic cells (DCs), sEVs contain proteins, lipids, mRNAs, noncoding RNAs (ncRNAs), and other molecules with a density ranging from 1.10 to 1.20 g/mL.^14^ The contents of cancer‐derived sEVs are innately heterogeneous and may exert powerful effects on recipient cells.^16^

sEVs have been isolated from bodily fluids, for example, plasma, plural effusion, breast milk, saliva, tears, and urine. Numerous sEVs isolation and enrichment techniques have been developed; however, nonvesicular molecular structures inevitably contaminate the isolated sEVs. The principal conventional methods used for isolation and enrichment are “standard” ultracentrifugation, gradient centrifugation, polymer‐based precipitation, size‐exclusion chromatography, and immunoaffinity chromatography, with the advantage of high throughput. On the other hand, recently developed methods, such as microfluidic filtering, contact‐free sorting, and immunoaffinity enrichment, have increased the enrichment efficiency and specificity, but at a lower throughput. Overall, the advantages and disadvantages of isolation methods exist objectively in both conventional and novel techniques, and thus further studies are needed to develop methods for more efficient isolation of sEVs.^14^ To date, a consensus “gold‐standard” isolation method has not been identified, and the method basically depends on the downstream applications and specific scientific questions.

Although “sEVs‐specific” markers are not currently available, investigators have reported at least three positive protein markers in a semiquantitative manner, such as Western blotting or flow cytometry, to generally characterize sEVs. Positive proteins include at least one transmembrane/lipid‐bound protein (CD9 and CD63) and one cytosolic protein (TSG101). In addition, the levels of proteins that are not expected to be enriched, such as calnexin, should also be determined. Moreover, the characterization of single vesicles in a mixture is recommended to provide an indication of the heterogeneity. At least two different but complementary techniques should be utilized for characterization, such as electron microscopy (transmission) or atomic force microscopy.^17^, ^18^ Furthermore, several sEVs and EV databases provide lists of the contents that have been identified, and researchers could compare the components they have isolated with the components listed in the databases EVpedia, Vesiclepedia, Exocart, exoRBase, and EVmiRNA.^19^, ^20^, ^21^, ^22^, ^23^

## 3 CRC sEVs AND METASTASIS

Patients with CRC tend to experience a high death rate due to metastasis or tumor recurrence.^24^ Based on emerging evidence, sEVs critically mediate CRC metastasis by transporting biomolecules between different cell types and play a pivotal role in the intricate process of tumor biofunction. Therefore, it is imperative to determine how sEVs exert their effects on the metastasis of CRC.

### 3.1 sEVs and cell proliferation, invasion, and migration in CRC

Some signaling pathways are engaged in the process of CRC formation, of which the canonical Wnt‐β‐catenin pathway is ubiquitously active in CRC development, and the most prevalent genetic events are mutations that disrupt the Wnt signaling cascade.^25^ Simultaneously, different Wnts behave in a similar manner in terms of the biochemical signaling pathways or effects on target cells, and β‐catenin is crucial effector of the pathway.^26^ EVs recruiting mutant β‐catenin might activate the Wnt signaling pathway to stimulate proliferation and migration of recipient cells^27^; meanwhile, hypoxic CRC cell‐derived sEVs simultaneously shuttle Wnt4 to normoxic cells to promote invasion and migration by stimulating the Wnt signaling pathway.^28^ Overexpression of cancer‐associated fibroblast (CAF)‐derived sEVs‐miR‐92a‐3p downregulates the expression of its target genes FBXW7 and MOAP1 to inhibit the ubiquitination and degradation of β‐catenin, resulting in the invasion and migration of CRC cells by activating the Wnt‐β‐catenin signaling pathway.^29^ According to Liu et al, sEVs‐miR‐486‐5p, an oncogene, directly binds to PLAGL2 to stimulate CRC cell growth and migration by inducing the expression of genes involved in the IGF2‐β‐catenin signaling pathway.^30^ The tumor‐suppressor gene APC ordinarily negatively regulates the canonical Wnt signaling pathway; nevertheless, in another pathway, APC downregulates PPARα binding to the lncRNA‐APC1 promoter to increase lncRNA‐APC1 expression, which suppresses CRC cell proliferation and metastasis by inhibiting sEVs production.^31^, ^32^

**FIGURE 2:**
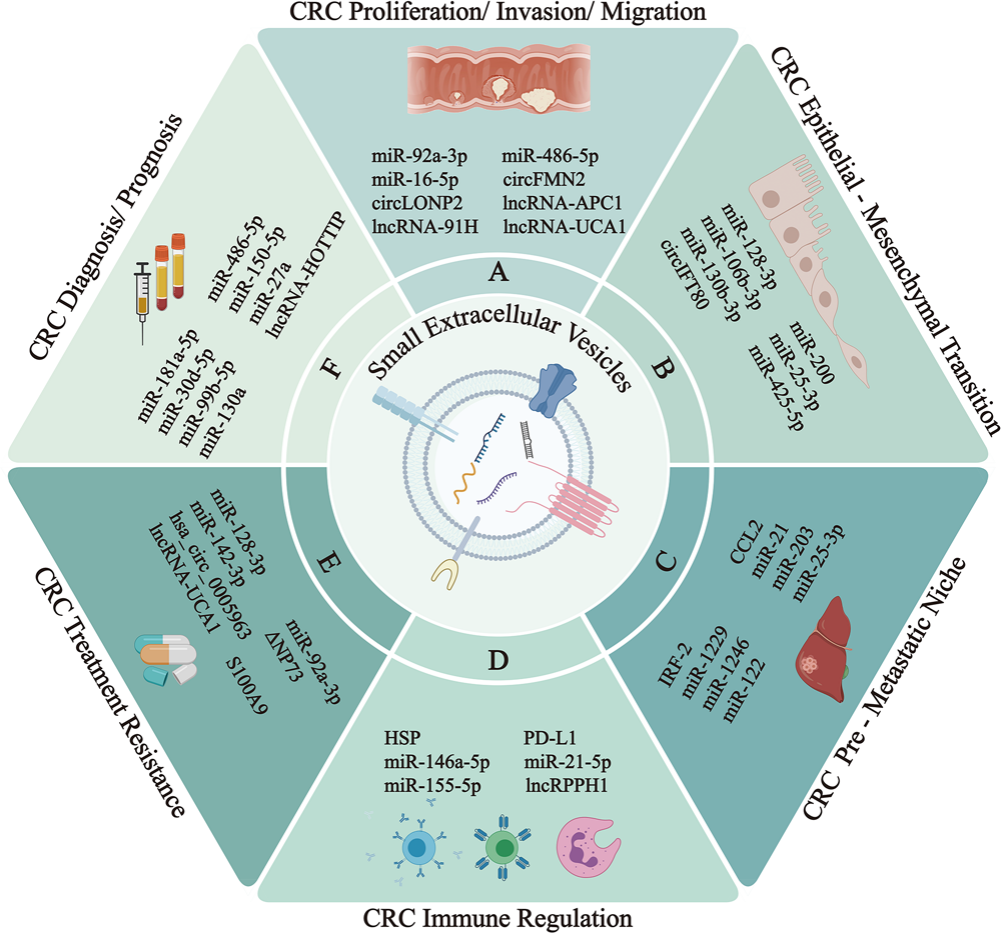
sEVs play a significant role in colorectal cancer proliferation/invasion/migration (A), epithelial‐mesenchymal transition (B), premetastatic niche (C), immune regulation (D), treatment resistance (E), and diagnosis/prognosis (F)

Additionally, numerous sEVs biomolecules are involved in a special signaling axis or signaling targets to alter tumor metastasis. Notably, miR‐16‐5p contained in sEVs derived from bone marrow‐derived mesenchymal stem/stromal cells (BMSCs) functions as a tumor suppressor that inhibits the proliferation, invasion, and migration and simultaneously promotes the apoptosis of CRC cells by reducing the expression of integrin α2 (ITGA2).^33^ The levels of sEVs‐lncRNAs and circRNAs are associated with the regulation of metastasis and proliferation in CRC.^34^, ^35^, ^36^ High sEVs‐lncRNA 91H levels promote invasion and migration by interacting with and modifying the expression of the RNA‐binding protein HNRNPK.^37^ The oncogenic lncRNA UCA1 increases the proliferation, apoptosis, and metastasis of CRC cells both in vitro and in vivo. Furthermore, UCA1 regulates MYO6 expression by directly sponging miR‐143.^38^ CircFMN2, which is abundant in serum sEVs, sponges miR‐1182 to eventually increase the expression of hTERT and substantially accelerate CRC cell proliferation.^39^ Nevertheless, circLONP2, which is mainly located in the nucleus, has a vastly different function from conventional sEVs circRNAs. It serves as a vital metastasis‐initiating factor that promotes CRC invasion and metastasis in distant organs by promoting the maturation and packing of miR‐17 into cell‐derived sEVs.^40^ As described above, these studies definitely illustrate that ncRNAs packed in sEVs exert important effects on recipient CRC cells and regulate their proliferation, migration, invasion, and metastasis (Figure 2A).

### 3.2 sEVs and the epithelial‐mesenchymal transition (EMT) of CRC

EMT is characterized by a loss of epithelial properties (ie, low levels of E‐cadherin and β‐catenin) and a gain of mesenchymal properties (ie, high levels of N‐cadherin and vimentin), and plays a predominant role in metastasis and drug resistance, and therefore is associated with a poor prognosis.^41^ Accumulating evidence has revealed the association between sEVs and EMT.

Notably, some miRNAs suppress the EMT in CRC. For instance, sEVs containing a high level of miR‐128‐3p appear to selectively transfer miR‐128‐3p to oxaliplatin‐resistant CRC cells, resulting in the inhibition of the oxaliplatin‐induced EMT.^41^ The miR‐200 family (miR‐200a, 200b, 200c, 141, 429) contained in the sEVs of CRC cells is transferred to blood and lymph endotheliocytes to further repress the EMT of endothelial cells.^42^, ^43^ In contrast, many miRNAs function to promote the EMT. High expression levels of miR‐106b‐3p,^44^ miR‐25‐3p, miR‐130b‐3p, and miR‐425‐5p^45^ promote the EMT through various pathways, and miR‐25‐3p facilitates the EMT by activating the canonical PTEN‐PI3K‐Akt‐STAT6 signaling axis. Circular RNAs contained in sEVs might function as competing endogenous RNAs (ceRNAs) to regulate the EMT in patients with mCRC. For example, sEVs‐circIFT80 functions as a ceRNA of miR‐1236‐3p to further regulate HOXB7 expression, which subsequently promotes the EMT of CRC^46^ (Figure 2B).

### 3.3 sEVs and the premetastatic niche (PMN) of CRC

The survival rate of patients with CRC substantially decreases to approximately 12% when distant organ metastasis occurs, increasing the importance of identifying critical components involved in the process of tumor metastasis and exploring new strategies to prevent tumor metastasis.^47^, ^48^ Based on Paget's “seed‐and‐soil” theory, a novel concept of the PMN was proposed to elucidate mechanisms of tumor metastasis from primary sites to secondary or remote organ sites.^49^ The PMN is a microenvironment that primarily consists of tumor‐derived cytokines, growth factors, sEVs, immune cells, and host stromal cells, and prepares a suitable site for the dissemination and growth of circulating tumor cells (CTCs).^50^, ^51^ Liu and Cao proposed six characteristics to define the PMN that promote metastasis and enable colonization, including vascular leakiness and angiogenesis, lymphangiogenesis, inflammation, immunosuppression, reprogramming, and organotropism.^52^ As components of the PMN, tumor‐derived sEVs were recently proposed to regulate the formation of the PMN in specific organ sites.^53^, ^54^ Therefore, we desire to obtain insights into how sEVs regulate the formation of the PMN in CRC (Figure 2C).

#### 3.3.1 Vascular leakiness and angiogenesis

**FIGURE 3:**
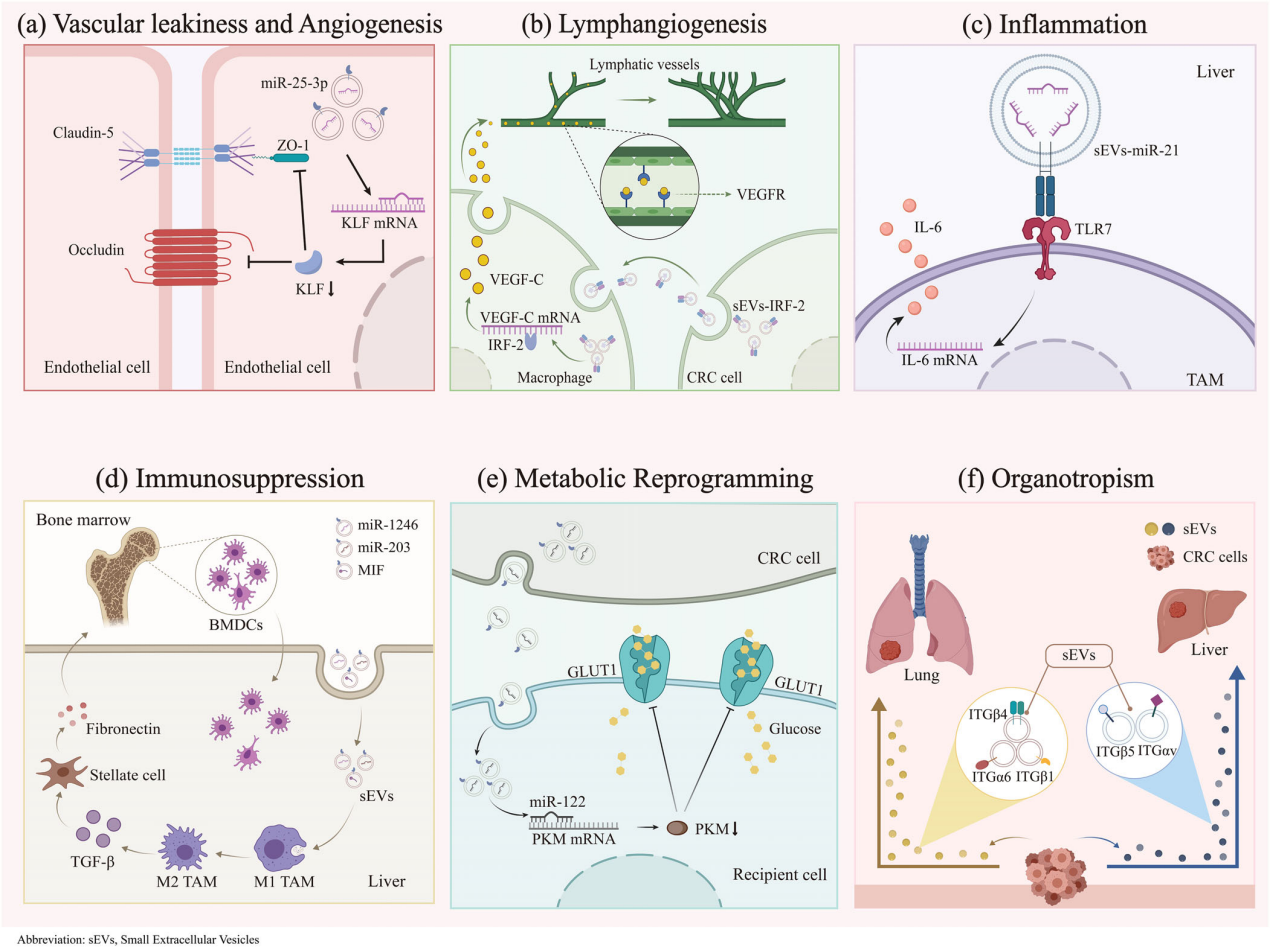
The mechanisms of sEVs in the formation of premetastatic niche in colorectal cancer. A, *Vascular leakiness and angiogenesis*: sEVs‐miR‐25‐3p induces vascular leakiness and angiogenesis by downregulating KLF family, ZO‐1, occludin, Claidin5, and VEGFR2. B, *Lymphangiogenesis*: sEVs‐IRF‐2 stimulates VEGF‐C secretion by sentinel lymph node (LN) macrophages to promote lymphangiogenesis. C, *Inflammation*: sEVs‐miR‐21 polarizes liver macrophages via miR‐21‐TLR7‐IL6 axis, which induces chronical inflammation. D, *Immunosuppression*: sEVs‐miR‐1246, miR‐203, and MIF polarize macrophages to increase TGF‐β expression, and then activate hepatic stellate cells to secrete fibronectin, which recruits bone marrow‐derived dendritic cells (BMDCs) to promote immunosuppressive microenvironment. E, *Reprogramming*: sEVs‐miR‐122 inhibits PKM expression to reduce GLUT1 and glucose uptake to reprogram the metabolism. F, *Organotropism*: sEVs‐ITGα_6_/ITGβ_4_/ITGβ_1_ are enriched in lung tropic sEVs, while sEVs‐ITGβ_5_/ITGα_v_ were primarily liver tropic

The vascular endothelial cell layer is connected by adherens and tight junctions, which provides a physical barrier to cells and body fluids. Tumor‐derived sEVs impair the functions of EC junctions to promote vascular permeability for further CTC entry into specific sites of distant organs.^49^, ^55^, ^56^ CRC‐derived sEVs‐miR‐25‐3p contributes to the induction of vascular leakiness and angiogenesis by downregulating the expression of the Krüppel‐like factor (KLF) family (zinc finger‐containing transcription factors), followed by the inhibition of the downstream endothelial cell targets ZO‐1, occludin, Claudin5, and VEGFR2. Consequently, sEVs‐miR‐25‐3p was capable of impairing the junctions of the endothelial cell layer, which increased the formation of the PMN and CRC metastasis in liver and lung in vivo.^57^, ^58^ Moreover, CRC cells delivered miR‐1229 into vascular endothelial cells to induce VEGF expression and subsequently promote angiogenesis^59^ (Figure 3A).

#### 3.3.2 Lymphangiogenesis

Lymphatic vessels probably serve as a primary point of access for the lymphatic dissemination of tumor cells,^60^ and lymphangiogenesis precedes CTC arrival at distant organ sites.^61^, ^62^ Therefore, lymphangiogenesis is actively involved in the formation of the PMN. According to clinical data, tumor‐derived VEGFs promote premetastatic lymphangiogenesis in regional lymph nodes (LNs).^63^ CRC‐derived sEVs‐IRF‐2 (interferon regulatory factor 2) has been postulated to stimulate VEGF‐C secretion by sentinel LN macrophages, resulting in lymphangiogenesis and metastasis^64^ (Figure 3B).

#### 3.3.3 Inflammation

Chronic inflammation is a critical driver of tumor progression and metastasis, and thus the local inflammatory microenvironment is an important factor contributing to the formation of the PMN. As components of the microenvironment, sEVs are involved in regulating inflammation by facilitating the infiltration of inflammatory cells.^52^ Notably, inappropriate activation of Toll‐like receptor 4 (TLR4) or other signaling pathways in immune cells may lead to unexpected inflammation.^65^ CRC‐derived sEVs‐miR‐21 is capable of polarizing liver macrophages into an IL‐6‐secreting phenotype by binding to TLR7 in tumor‐associated macrophages (TAMs), which contributes to the creation of an inflammatory PMN.^47^ Thus, the miR‐21‐TLR7‐IL6 axis would be a potential therapeutic target for patients with CRC that has metastasized to the liver (Figure 3C).

#### 3.3.4 Immunosuppression

The recruitment of immune cells to establish an immunosuppressive microenvironment is a hallmark of PMN formation. In particular, bone marrow‐derived dendritic cells (BMDCs) are one of the main effector cells that suppress the antitumor response and allow primary tumor cells to overcome the immune defenses.^66^ sEVs containing large amount of miR‐1246 that are derived from TP53 mutant CRC cells, miR‐203‐enriched sEVs and macrophage migration inhibitory factor‐containing sEVs originating from CRC cells are taken up by liver macrophages, resulting in the polarization of macrophages to M2 TAMs to increase transforming growth factor‐β (TGF‐β) levels,^47^, ^67^ subsequently inducing neighboring hepatic stellate cells to secrete fibronectin, and ultimately contributing to the recruitment of BMDCs that promote PMN formation.^68^ Interestingly, a classical Chinese medicine, Dahuang Zhechong pill, reduces the expression of sEVs‐CC chemokine ligand‐2 (CCL2) to repress TAM recruitment and inhibit the polarization of M1 cells to a M2 phenotype, eventually ameliorating the formation of the PMN^69^ (Figure 3D).

#### 3.3.5 Reprogramming

Stromal, metabolic, and epigenetic reprogramming are engaged in PMN‐induced tumor metastasis. As shown in recent studies, sEVs are associated with recruiting and reprogramming host stromal cells, such as fibroblasts and epithelial cells, into the PMN to modify the PMN.^70^, ^71^ sEVs‐miR‐122 taken up by recipient cells specifically inhibits pyruvate kinase (PKM) expression to reduce glucose transporter 1 levels and glucose uptake, which reprogrammed the metabolism of PMN to facilitate metastasis^72^ (Figure 3E). sEVs‐contained miRNAs may epigenetically exhaust the expression of phosphatase and tensin homolog deleted on chromosome ten (PTEN) to induce the secretion of chemokine CCL2, subsequently recruiting tumor‐promoting myeloid cells to promote PMN formation.^73^ Regrettably, few studies have examined how sEVs facilitate the reprogramming of PMN in CRC; therefore, further investigations assessing how remodeling occurs are urgently needed.

#### 3.3.6 Organotropism

The concept of organotropism is described as the ability of certain tumors to metastasize to specific organs. Tumor‐derived sEVs express particular integrins (ITGs) that interact with extracellular matrix molecules (laminin and fibronectin) to initiate the formation of PMN in a tissue‐specific manner. ITGα_6_/ITGβ_4_/ITGβ_1_ are enriched in lung‐tropic sEVs, while in parallel, ITGβ_5_/ITGα_v_ are primarily enriched in liver‐tropic sEVs^4^ ^9^ (Figure 3F). In a CRC model, integrin beta‐like 1 activated CAFs in remote organs through the TNFAIP3‐NF‐κB signaling pathway, and subsequently the stimulated CAF secretion of the proinflammatory cytokines IL‐6 and IL‐8 to promote formation of the PMN.^74^

In summary, all these studies have undoubtedly provided a foundation of further investigations of the role of sEVs in PMN formation and propose a promising strategy to prevent metastasis of CRC.

## 4 CRC sEVs AND IMMUNE REGULATION

Cancer‐derived sEVs are mechanistically engaged in impairing an effective immune response by modulating the maturation and antitumor activity of immune cells,^75^, ^76^ which promotes the establishment of an immunosuppressive microenvironment for cancer cells.^77^ Programmed cell death ligand‐1 (PD‐L1) expressed on the surface of cancer cells binds its receptor PD‐1 on effector T cells, thus attenuating their activity in antitumor immunity; not surprisingly, PD‐L1 is also present on the surface of sEVs. In a Rab27a knockout CRC model, PD‐L1^+^ sEVs appeared to inhibit T‐cell activity to promote tumor growth and resist to immune checkpoint protein inhibitors.^78^

CD8^+^ T cells represent the chief antitumor effector cells in the tumor microenvironment (TME). However, the dysfunction of CD8^+^ T cells impairs the antitumor effect.^79^ Cheng and colleagues revealed that sEVs‐miR‐146a‐5p, a major miRNA expressed in cancer stem cells (CSCs) that depends on Rab27a activation, decreased the number of tumor‐infiltrating CD8^+^ T cells, thus promoting the formation of an immunosuppressive cancer microenvironment in CRC.^80^ CRC sEVs induced a shift in the phenotype T cells to tumor‐supporting Treg‐like cells by activating TGF‐β‐Smad signaling and inactivating SAPK signaling.^81^ In addition to impairing T‐cell function, Huber et al proposed that CRC cells release numerous sEVs containing Fas‐ligand positive and tumor necrosis factor‐related apoptosis‐inducing ligands to promote CD8^+^ T‐cell apoptosis, thus creating an immunosuppressive microenvironment.^82^

Generally, M2 TAMs, which are known as alternatively activated macrophages, are required to promote tumorigenesis by secreting pro‐tumor factors, such as inflammatory cytokines, chemokines, and angiogenic factors.^83^, ^84^ sEVs are capable of inducing macrophages to differentiate into the pro‐tumor M2 phenotype.^85^ LncRPPH1‐loaded sEVs, which are present at high levels in serum, are transferred into macrophages to mediate macrophage M2 polarization, which in turn promotes the proliferation and metastasis of CRC cells.^86^ Furthermore, Lan et al have reported that M2 macrophage‐derived sEVs containing miR‐21‐5p and miR‐155‐5p downregulate the expression of BRG1 by directly binding to the BRG1 coding sequence in CRC cells, thus resulting in the metastasis of CRC.^87^

DCs are potent antigen‐presenting cells that activate T cells to induce an antitumor response. However, EVs derived from cancer cells are able to block DC activity through various signaling pathways.^88^ One study reported an obvious upregulation of TNF, TGF‐β, and IL‐6 in DCs cocultured with EVs, which subsequently decreased their phagocytic activity, suppressed the proliferation of T cells, and impaired the cytolytic potential of T cells by downregulating intracellular granzyme B, perforin, and IFN‐γ.^89^ sEVs‐heat shock protein (HSP) derived from B lymphoma cells more efficiently induces both the phenotypic and functional maturation of DCs, and simultaneously stimulates the antitumor activity of CD8^+^ T cells.^90^

Although neutrophils are characterized by neutral staining, the role of neutrophils in cancers is by no means neutral. Neutrophils possess both tumor‐promoting and tumor‐suppressing functions, depending on numerous factors,^91^ including polarization (N1 or N2 phenotype) and location relative to the tumor (intratumor, peritumor, or stromal).^92^, ^93^ CSC sEVs transported to bone marrow not only increase neutrophil survival but also reprogram these cells to the pro‐tumor phenotype that secretes IL‐1β, mainly by activating the NF‐κB signaling pathway.^94^ Recently, the role of neutrophil extracellular traps (NETs), the extracellular mesh‐like structures containing DNA and cytosolic granular proteins, released from neutrophils in cancer have attracted increasing attention.^95^ Specifically, sEVs derived from KRAS mutant CRC induce neutrophil accumulation and the formation of NETs to promote CRC growth and metastasis, and the effect was abolished by an anti‐IL‐8 treatment.^96^, ^97^, ^98^ This evidence revealed roles for sEVs in the survival, migration, phenotype transition, and NET release of neutrophils.

The studies described above reveal the functions of sEVs in inhibiting antitumor immunity; however, the exact role of sEVs is still a topic of debate. Limited studies have been conducted to elucidate the antitumor effect of sEVs. HSP on the surface of sEVs from CRC cells induces the migration and cytolytic activity of natural killer (NK) cells following the release of granzyme B to initiate tumor apoptosis.^99^ The other pathway of HSP is to inhibit tumor growth by converting immunosuppressive regulatory T cells to Th17 cells via IL‐6.^100^ Taken together, further research is indeed needed to understand the mechanisms by which sEVs regulate the immune response in CRC, and to identify potential prognostic factors and antitumor therapies (Figure 2D).

## 5 CRC sEVs AND TREATMENT RESISTANCE

Important achievements in mCRC treatment have been reported, such as the application of conventional therapeutics based on 5‐fluorouracil with oxaliplatin or irinotecan, monoclonal antibodies such as panitumumab and cetuximab,^24^, ^101^, ^102^, ^103^ and pre‐ and postoperative radiotherapy. However, a large number of patients exhibit different levels of treatment resistance, which directly results in reduced survival rate.^104^ Therefore, the mechanisms of primary or acquired therapy resistance must be clarified to improve the survival rate of patients with mCRC. Many researchers have analyzed sEVs released by stromal or cancer cells and their potential pivotal roles in treatment resistance^105^ (Figure 2E).

### 5.1 sEVs and drug resistance

The conventional mechanisms of drug resistance have been elucidated by numerous researchers over the past few decades, including mutations in p53, overexpression of ATP‐binding cassette efflux transporters, alterations in cellular drug influx/efflux, disruption of apoptotic pathways, and single‐nucleotide polymorphisms in platinum or fluoropyrimidine targets, among others.^104^, ^106^

In addition to the mechanisms mentioned above, scholars’ interest has shifted to the exploration of cell‐derived sEVs. Two major monoclonal antibodies, cetuximab and panitumumab, directly bind to the extracellular domain of the epidermal growth factor receptor (EGFR) and admittedly improve the survival of patients with mCRC expressing wild type RAS, including improvements in overall survival, progression‐free survival, and response rate, both as single agents and in combination with chemotherapy.^107^, ^108^ EGFR is an essential component of the pathway regulating cell proliferation by activating several principal downstream pathways, the RAS‐RAF‐MAPK, PI3K‐PTEN‐AKT, and JAK‐STAT pathways, and mutations in any protein involved in these pathways, such as KRAS, BRAF, and PIK3CA, may lead to resistance to anti‐EGFR therapy.^109^ sEVs derived from cetuximab‐resistant cells have been shown to “infect” cetuximab‐sensitive cells and transform them to cetuximab‐resistant cells by reducing the expression of PTEN and increasing the levels of phosphorylated AKT.^110^ Although the specific role of sEVs was not clearly determined in this previous study, another study revealed that sEVs‐lncRNA UCA1 is essential to confer cetuximab resistance in CRC cells by participating in RNA‐RNA interactions.^111^ Overexpression of UCA1 contributes to reducing the expression of miRNA targets, which induces the deregulation of certain signaling pathways.^112^ Nevertheless, overexpression of sEVs‐transmitted miR‐128‐3p was recently shown to increase the chemosensitivity of oxaliplatin‐resistant cells by inhibiting the expression of the drug transporter MRP5 to reduce oxaliplatin efflux.^113^

CSCs are characterized by the surface markers CD133 and CD44,^114^ self‐renewal, and the ability to differentiate into various cell types, which are inherently chemoresistant and enriched in recurrent cancer.^115^, ^116^, ^117^, ^118^ Therefore, several researchers have elaborated the association of sEVs with the formation of CSCs and resistance to chemotherapy. sEVs derived from CAFs consist of different types of RNAs, and the direct transfer of sEVs‐miR‐92a‐3p to CRC cells primes stem cells and induces chemoresistance by activating the Wnt‐β‐catenin pathway and inhibiting FBXW7 and MOAP1.^29^, ^118^, ^119^ sEVs derived from myeloid‐derived suppressor cells maintain the stemness of CRC cells by delivering S100A9 to bind and activate NADPH oxidase, which activates NF‐κB and STAT3.^115^ sEVs from BMSCs carry miR‐142‐3p, which increases the population of CSCs by downregulating the protein Numb (inhibitor of the Notch signaling pathway), thus activating the Notch signaling pathway.^120^ However, miR‐142‐3p may also inhibit the stemness of CRC cells by targeting CD133, Lgr5, and ABCG2. Therefore, the role and mechanisms of miR‐142‐3p require further study.^121^

Cytotoxic drugs are very important treatments for patients with mCRC and are often administered in combination with other anticancer drugs. The sEVs‐circular RNA hsa_circ_0005963 delivered from oxaliplatin‐resistant cells is transferred to chemosensitive cells, increasing glycolysis to induce drug resistance by regulating the miR‐122‐PKM2 axis.^122^ Moreover, ΔNP73, an isoform of homolog of p53, is transferred by sEVs to confer resistance to oxaliplatin, thus promoting CRC cell proliferation.^123^

Overall, drug resistance exists, and an effective method is not available to address this problem. Additionally, sEVs represent a specific focus area for researchers to elucidate the mechanisms of drug resistance and improve treatments for patients with mCRC.

### 5.2 sEVs and radiotherapy resistance

Pre‐ and postoperative radiotherapy prolonged disease‐free survival in several randomized trials,^124^, ^125^, ^126^ of which preoperative radiotherapy achieved better effects.^127^ However, similar to resistance to chemotherapy, radioresistance occurs and the response rate is only approximately 20%.^128^ A resistance mechanism related to CAF‐derived sEVs has been identified, as CAF‐derived sEVs stimulate the TGF‐β signaling pathway to program CRC cells to the CSC phenotype characterized by clonogenicity and radioresistance.^129^ Studies on this topic are extremely limited; accordingly, the underlying mechanisms of radiotherapy resistance must be elucidated to provide better therapeutic strategies in the future.

## 6 CRC sEVs AND CLINICAL THERAPY

As described above, drug resistance is quite common in CRC. With the characteristics of high biocompatibility, low immunogenicity, and efficient delivery, sEVs have become a potential drug delivery system.^130^, ^131^ Early in 2008, 40 patients with advanced CRC were enrolled in a phase I clinical trial and were treated with ascites‐derived sEVs in conjunction with the granulocyte‐macrophage colony‐stimulating factor, but therapeutic responses were rarely detected, with the exception of a few patients in the stable state.^132^ Recently, the University of Louisville designed a randomized phase I clinical trial to investigate the ability of plant sEVs to deliver curcumin to normal and malignant colon tissues (NCT01294072) that was based on the biocompatibility of sEVs to increase the stability, solubility, and bioactivity of curcumin in cancer cells and immune cells in colon cancer.

Some researchers have postulated that sEVs loaded with drugs, coated with certain high‐density antibodies, and functionalized with targeting ligands would display an improved tumor‐targeting ability and inhibit cell proliferation, which might represent an innovative delivery system for drugs targeting specific cancers.^133^ Deregulated miRNA expression is associated with the chemotherapy resistance pathway, and thus the inhibition of oncogenic miRNAs represents a potential therapeutic strategy.^134^ According to Liang et al, the co‐delivery of 5‐fluorouracil and a miR‐21 inhibitor by engineered sEVs reverses anticancer drug resistance in CRC.^135^ As shown in another study, miR‐128‐3p is crucial regulator that increases intracellular oxaliplatin accumulation to increase the chemosensitivity of CRC.^113^

## 7 CRC sEVs AND FUTURE PERSPECTIVES IN THE CLINIC

Early detection of CRC would contribute remarkably to improving the prognosis and survival of patients. With the increasing application of colonoscopy in the past few decades, the detection rate has increased. However, the invasive process is likely to create complications, such as pain, intestinal perforation, or bleeding, in patients.^136^, ^137^ Currently, “liquid biopsy,” an easy, noninvasive, reproducible, economic, and acceptable strategy compared to traditional biopsy, has been shown to be potential complement to surgical biopsy in the diagnostic screen and monitoring of the patient's condition. The assays of the molecules and cells present in the “liquid biopsy” include circulating tumor DNAs, CTCs, tumor‐educated platelets, circulating free RNAs, circulating miRNAs, and circulating sEVs.^138^ Among these markers, sEVs are characterized by stability (the constituents are protected from degradation by the phospholipid bilayer membrane) and ready availability (abundant in the blood), which make them relatively promising biomarkers in clinical practice^139^ (Figure 2F).

With the assistance of bioinformatics, a group systematically analyzed the diagnostic value of CRC‐associated sEVs long RNAs, and revealed that the combination of KRTAP5‐4, MAGEA3, and BCAR4 exhibited promise in distinguishing colorectal adenoma and cancer from the healthy tissue, suggesting a possible strategy for detecting early‐stage CRC.^140^ Another study provided additional evidence that sEVs‐lncRNAs (LNCV6_116109, LNCV6_98390, LNCV6_38772, LNCV_108266, LNCV6_84003, and LNCV6_98602) are upregulated in CRC, particularly in patients with stage I‐II tumors, and these molecules might also represent early diagnostic biomarkers for CRC.^3^

Bjørnetrø and colleagues identified sEVs‐miRNAs originating from hypoxic tumor cells as circulating biomarkers to predict high‐risk locally advanced rectal cancer (LARC), of which miR‐181a‐5p, miR‐486‐5p, and miR‐30d‐5p were associated with LN metastasis, organ‐invasive primary tumors, and metastasis progression, respectively.^141^ The lncRNA HOTTIP not only identifies patients with CRC but also potentially functions as a valuable surrogate biomarker for presurgical risk stratification, as HOTTIP expression significantly predicted the survival time after surgery.^142^ A clinical trial (NCT03874559) aims to characterize the levels of sEVs biomarkers in patients with LARC undergoing neoadjuvant chemoradiation therapy to compare the rates of sEVs biomarker expression before, during, and after chemoradiation therapy, which may identify specific prognostic biomarkers.

Interestingly, some biomolecules possess both diagnostic and prognostic properties. Circulating sEVs‐miR‐150‐5p, miR‐99b‐5p, miR‐27a, and miR‐130a levels might serve as novel diagnostic and prognostic biomarkers for patients with CRC, as sEVs‐miR‐150‐5p and miR‐99b‐5p are downregulated, whereas miR‐27a and miR‐130a are upregulated.^143^, ^144^

Several clinical trials (NCT03874559; NCT04227886; NCT04394572; NCT04523389) aim to characterize sEVs‐contained biomarkers to help identify specific diagnostic and/or prognostic biomarkers for CRC. NCT03874559 and NCT04227886 both aim to characterize the levels of sEVs biomarkers in patients with LARC undergoing neoadjuvant chemoradiation therapy to compare the rates of sEVs biomarker expression before, during, and after chemoradiation therapy, which may identify specific prognostic biomarkers. To step further, NCT04394572 specifically screens sEVs protein markers for CRC diagnostic and/or prognostic, while NCT04523389 wants to figure out specific sEVs miRNAs for early CRC prognosis. Though clinical trials are still under progress, once completed, they could provide an insight into sEVs‐related biomarkers to help early diagnosis and prognosis for CRC patients.

## 8 DISCUSSION

CRC is one of the most common and prevalent malignant cancers, with high morbidity and mortality rates being reported worldwide. The patient's condition is exacerbated as a result of a delayed diagnosis, therapy resistance, and poor prognosis. Characterized by lipid bilayer membrane and carrying a number of biomolecules, sEVs are now publicly recognized to serve as messengers involved in intercellular communication in the TME and to participate in the progression of CRC. As is summarized above, we mainly discuss how sEVs‐related molecules action to recipient cells that contributes to CRC progression, metastasis, and other potential clinical applications. Studies aiming to elucidate the detailed mechanisms would help identify potential targets to block tumor progression.

Although numerous researchers have attempted to determine the mechanism by which sEVs modulate the pathogenesis of CRC, several challenges and obstacles in EVs field cannot be ignored. As proposed, there are various kinds of isolation methods for EVs isolation; however, due to limitations of isolation and characterization methods, it is still unrealistic to propose specific markers to classify each type of EVs. Besides, when function to recipient cells of EVs mentioned in research articles, we should be aware of that some non‐EVs components often coisolate with EVs, such as albumin, soluble immune active cytokines, or even platelets from plasma, which contaminates the EVs and might give wrong information of EVs function. Moreover, these contaminants may also influence the accuracy of sEVs‐based diagnosis. Therefore, when specific sEVs function is mentioned, it should be interpreted with caution. And it is important to choose and develop isolation techniques to isolate EVs with least soluble factor, which helps apply sEVs detection into clinical applications.^145^

Furthermore, there is still a debate whether plasma or serum is a better source of sEVs for clinical applications. Though plasma sEVs are deemed to contain more sEVs biomarkers and better for diagnosis than serum sEVs,^146^ plasma contains platelets that are able to release several types of membrane vesicles,^147^ which will confound the measurement of circulating miRNA biomarkers in plasma,^148^, ^149^ and even more nonvesicle miRNAs.^150^ Nevertheless, compared to plasma, sEVs originated miRNAs are more highly expressed in serum sEVs samples. Therefore, serum seems more preferable for cancer‐associated sEVs biomarker studies.^150^

In clinical application, it is important to give a specific dose of certain medical substance. Though there are many methods to quantify EVs such as quantification of particle number by nanoparticle tracking analysis, or measurement of particulate components such as proteins, lipids, and nucleic acids are relative suitable proxy for EVs quantification, it is still imperfect and need further improvement. Some work has tried to give solutions to this issue, and they build their own quantification platforms based on antibodies, immunoassay, and imaging flow cytometry, which are able to selectively detect tumor‐derived EVs.^151^, ^152^, ^153^ And in their work, compared to healthy donors, cancerous samples are able to release more EVs, and gene knockdown of organ‐tropic membrane proteins could decrease organ‐tropic EVs to specific organ.^154^ This might indicate that increasing of EVs secretion promotes cancer progression. Besides the quantification of sEVs, how sEVs contents are regulated also spawn our attention. Sumoylated sEVs‐hnRNPA2B1,^155^ ubiquitinated target proteins^156^ seem to be vital for molecules to be sorted into sEVs, which might associate with cancer metastasis. Moreover, a stoichiometric analysis of sEVs‐miRNA raised the awareness that the majority of individual sEVs do not carry sufficient miRNAs, with less than one copy of miRNA per sEVs.^157^ Therefore, the presence of heterogeneity of distinct subset of sEVs warrants the significance to establish specific markers of specific subset of sEVs, and further methods to isolate interested subsets of sEVs are needed.

Additionally, although some molecules were described to be latent biomarkers, more meaningful and specific biomolecules involved in CRC must be discovered to increase the accuracy of diagnosis and prognosis. In addition, more clinical trials are urgently needed to validate the application of sEVs as biomarkers for clinical screening and monitoring the patient's condition. The advances in applying sEVs in patients with CRC are still limited but have rapidly attracted attention, and sEVs are predicted to be successfully employed in clinical therapy and the diagnosis of early‐stage CRC in the future.

## AUTHOR CONTRIBUTIONS

Dawei Li and Ping Wei conceived and critically reviewed the structure of manuscript. Xuefeng He drafted and revised the manuscript. Xinyang Zhong and Zijuan Hu collected literatures and completed tables. Senlin Zhao researched on the background of the study. All the authors read and approved the final manuscript.

## CONFLICT OF INTEREST

The authors declare that there is no conflict of interest.
